# The benefits of early continuous renal replacement therapy in critically ill patients with acute kidney injury at high-altitude areas: a retrospective multi-center cohort study

**DOI:** 10.1038/s41598-023-42003-6

**Published:** 2023-09-09

**Authors:** Bowen Wang, Mengjia Peng, Hui Wei, Chang Liu, Juan Wang, Liheng Jiang, Fei Fang, Yuliang Wang, Yuandi Shen

**Affiliations:** 1 Intensive Care Center, General Hospital of Tibet Military Command, Lhasa, 850000 Tibet China; 2 Department of Emergency, General Hospital of Tibet Military Command, Lhasa, 850000 Tibet China; 3 Intensive Care Center, Hospital of Chengdu Office of People’s Government of Tibetan Autonomous Region, Chengdu, 610041 Sichuan China; 4 Intensive Care Center, People’s Hospital of Tibet Autonomous Region, Lhasa, 850000 Tibet China; 5 Department of Emergency, Naval Medical Center of PLA, Shanghai, 200052 China

**Keywords:** Continuous renal replacement therapy, Acute kidney injury, Acute kidney injury, Clinical trials

## Abstract

Severe hypoxia would aggravate the acute kidney injury (AKI) in high-altitude areas and continuous renal replacement therapy (CRRT) has been used to treat critically ill patients with AKI. However, the characteristics and outcomes of CRRT in critically ill patients at AKI in high altitudes and the optimal timing of CRRT initiation remain unclear. 1124 patients were diagnosed with AKI and treated with CRRT in the ICU, comprising a high-altitude group (n = 648) and low-altitude group (n = 476). Compared with the low-altitude group, patients with AKI at high altitude showed longer CRRT (4.8 vs. 3.7, *P* = 0.036) and more rapid progression of AKI stages (*P* < 0.01), but without any significant minor or major bleeding episodes (*P* > 0.05). Referring to the analysis of survival and kidney recovery curves, a higher mortality but a lower possibility of renal recovery was observed in the high-altitude group (*P* < 0.001). However, in the high-altitude group, the survival rate of early CRRT initiation was significantly higher than that of delayed CRRT initiation (*P* < 0.001). The findings showed poorer clinical outcomes in patients undergoing CRRT for AKI at high altitudes. CRRT at high altitudes was unlikely to increase the adverse events. Moreover, early CRRT initiation might reduce the mortality and promote renal recovery in high-altitude patients.

## Introduction

Acute kidney injury (AKI) is characterized by a sudden loss of kidney function and is closely associated with morbidity and mortality in critically ill patients, leading to massive waste of health resources each year^1–3^. A meta-analysis of 3,585,911 participants revealed that 20.0–31.7% of in-hospital patients had AKI, with an average pooled mortality rate of 23–49.4%^4,5^. Kidneys are more easily injured in high-altitude areas^6^. High-altitude inhabitants who have severe hypoxia and pulmonary hypertension are more likely to develop hyperuricemia, hypertension, and proteinuria, which is defined as “high-altitude renal syndrome”^7,8^. According to previous studies, people in high-altitude areas have worse kidney function and a higher prevalence of proteinuria than those at sea level, which is partly due to the effects of hyperuricemia on glomerular hemodynamics and tubular function^6,9^. The high altitude population also experiences both erythrocytosis and hypertension, which can lead to renal hypoperfusion and exacerbate the burden on the kidneys^10^.

Continuous renal replacement therapy (CRRT), an efficient extracorporeal system well recognized for body hemostasis and solute control, is one of the most common used modality for the treatment of AKI in the intensive care unit (ICU)^11,12^. However, current CRRT prescriptions at high altitudes are largely based on physicians’ experiences because CRRT at high altitudes may have different adverse and curative effects on AKI than those at low altitudes. On the one hand, previous studies found that long-term environmental hypoxia was an independent factor in coagulation dysfunction, hemostatic disorders and thrombocytopenia at high altitude^13–15^. Sufficient use of anticoagulants in CRRT, which is required to prevent circuit clotting to maintain filter performance, is more likely to increase bleeding risk and other adverse events at high altitudes^1,7^. On the other hand, emerging data have demonstrated that unwarranted CRRT initiation might result in prolonged CRRT durations and deteriorated kidney injury^16,17^. The optimal timing for CRRT initiation in high-altitude areas remains to be determined.

Although the prevalence of high-altitude renal syndrome has been widely reported in Tibet, data on CRRT of patients with AKI in this region is scarce^9,18^. Meanwhile, the curative effects, adverse effects, and reasonable strategies for CRRT at high altitudes need to be further investigated. In the current study, we aimed to compare the characteristics, outcomes, and adverse events of CRRT in critically ill patients with AKI in both the high-altitude and low-altitude groups. Furthermore, the survival rate and kidney-related outcomes of the early and delayed CRRT initiation groups were compared to determine the best timing to start CRRT for high-altitude patients with AKI.

## Methods

### Study design and participants

**Figure 1:**
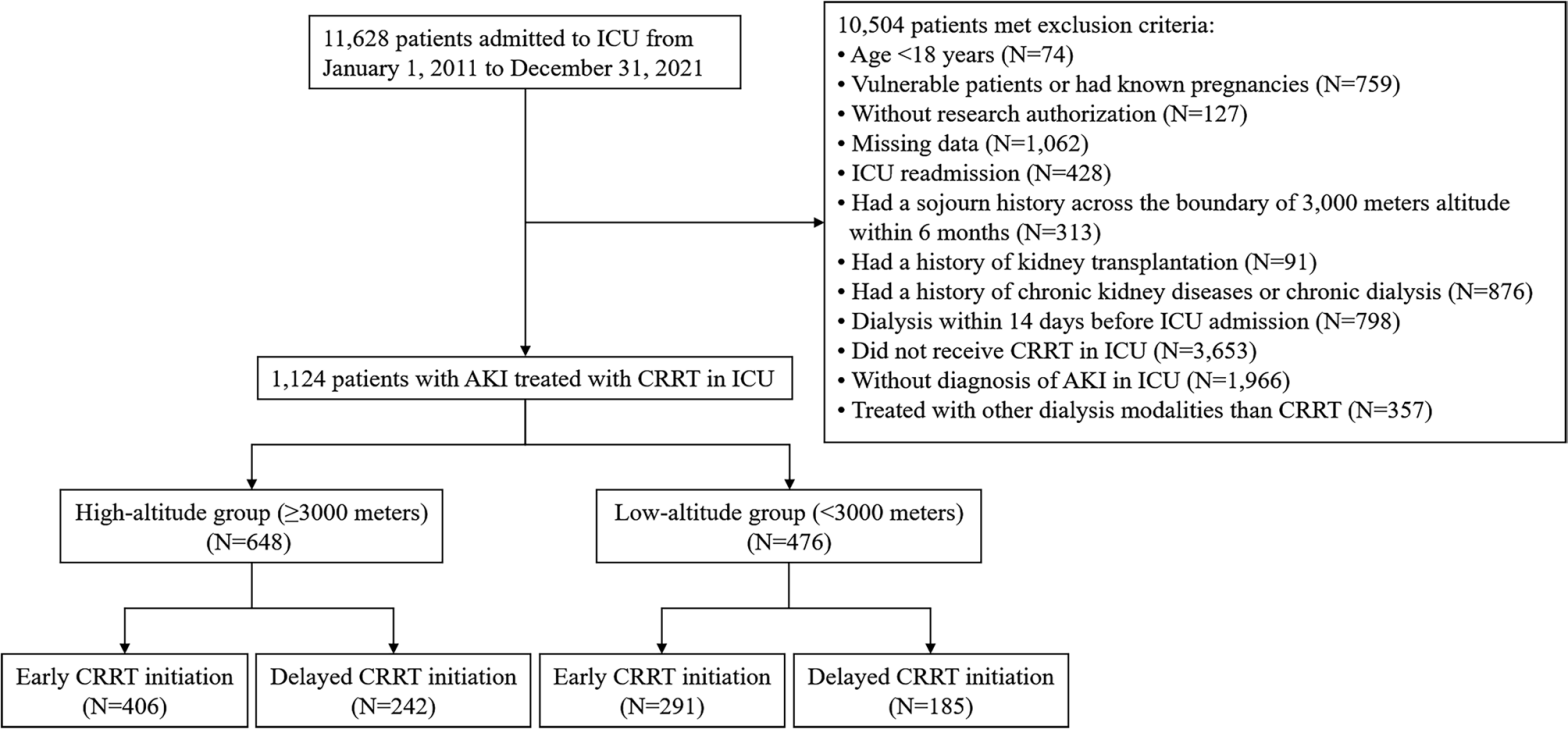
Flowchart of participants enrollment and grouping. *AKI* acute kidney injury, *CRRT* continuous renal replacement therapy, *ICU* intensive care unit.

This retrospective multi-center cohort study included three cohorts of participants from the General Hospital of Tibet Military Command in Lhasa, the People’s Hospital of Tibet Autonomous Region in Lhasa, and the Hospital of Chengdu Office of the People’s Government of the Tibetan Autonomous Region in Chengdu. According to the *International Statistical Classification of Diseases and Related Health Problems*, patients diagnosed with AKI and treated with CRRT in the ICU were retrospectively enrolled from January 1, 2011, to December 31, 2021, in the three hospitals listed above^17^. The exclusion criteria are shown in Fig. 1. This retrospective cohort study was approved by the Medical Ethics Committee of the General Hospital of Tibet Military Command of the Chinese People’s Liberation Army, and the requirement for informed consent was waived by the Ethics Committee because of its minimal risk. All the procedures performed in our study involving human participants with CRRT were in accordance with the ethical standards of the 1964 Helsinki Declaration.

### Case definitions

High-altitude patients were those who had lived at or above 3000 m for at least 10 years and had not visited places below 3000 m within 6 months. Low-altitude patients were those who had lived below 3000 m for at least 10 years and had not visited places at or above 3000 m within 6 months. The evaluation process for the patients’ altitude consisted of two steps: Firstly, we reviewed the individual histories in the electronic medical record system to ascertain if patients had a history of living or traveling to other regions. If patients had no travel history and their place of birth matched the location of their treatment, we recorded the altitude of their place of birth as their altitude. Secondly, if patients had a travel history or their place of birth differed from the treatment location, we contacted them or their family members via telephone. Only patients who explicitly confirmed that they did not have a sojourn history across the boundary of 3000 m were included in the analysis, while the remaining patients were excluded due to missing data. The average altitude of each region was obtained from the Center for Disease Control and Prevention (CDC) of the Tibet Military Command and verified using Google Earth. Different stages of AKI and acute kidney disease (AKD) were diagnosed according to *Kidney Disease: Improving Global Outcomes* (KDIGO)^19^. CRRT liberation was defined when the patient was in remission and CRRT was discontinued for more than 12 h, except that temporary treatment interruption was required for auxiliary examinations or surgery. For patients who had more than one CRRT liberation attempt in the ICU, only the first attempt was considered in this study. Kidney recovery was defined as AKD stage 0 being reached and maintained during hospitalization. AKD stage 0 referred to patients with an increase of serum creatinine level to less than 1.5 times the baseline level after CRRT initiation according to *KDIGO*^19^. Baseline creatinine levels defined as the mean of the outpatient creatinine values measured from 168 to 14 days before the admission. If there was no outpatient records, the baseline creatinine value of the patient was converted using the normal values, assuming normal baseline glomerular filtration rate of 60 mL/min per 1.73 m^2^^17^.

Early CRRT initiation referred to CRRT starting within the 24 h after the diagnose of AKI, while delayed CRRT initiation referred to CRRT starting more than 24 h after the diagnose of AKI^20,21^.

### CRRT prescription

The decisions regarding CRRT initiation, termination, or reinstitution for AKI patients were drawn by the specialized nephrology intensive care groups of the three hospitals. Nurses responsible for dialysis set up and checked the CRRT circuit on a regular basis, according to the intensive care physicians’ advice. Continuous venovenous hemodialysis (CVVHD) or continuous venovenous hemodiafiltration (CVVHDF), the standard modes of CRRT, were performed in Multifiltrate (Fresenius, Germany) with a 1.4 m^2^ membrane (AV 600S, Fresenius) or PRISMA (Gambro, Sweden) with a 0.9 m^2^ membrane (M100, Gambro). For CRRT vascular access, a temporary double-lumen dialysis catheter was implanted into the internal jugular or femoral vein.

The standard settings for CRRT prescription were listed following: (1) bicarbonate-buffered solution was used for the preparation of dialysis and hemofiltration; (2) blood flow rate was set to 180 ml/min; (3) the dialysis dose was set to 30 ml/kg of body weight/h; (4) 50% of dialysis and 50% of hemofiltration were used when the mode of CVVHDF was performed; (5) the anticoagulant citrate dextrose solution formula A or unfractionated heparin was used for anticoagulation strategies unless the existence of contradictions; (6) for regional citrate anticoagulation, continuous intravenous calcium chloride was infused to maintain homeostasis with dose-adjustments based on the results of ionized calcium measured every 4 h after CRRT initiation; (7) for unfractionated heparin anticoagulation, the infusion of heparin was increased until the value of activated partial thromboplastin time (APTT) was 2.0 times greater than the normal level; (8) if anticoagulation-related adverse events occurred, the dose of anticoagulant citrate and unfractionated heparin was slightly altered by intensive care physicians.

### Data collection

The demographic, clinical, and laboratory data of the patients were extracted from electronic medical records using a data collection form including the CRRT application and kidney-related outcomes. Laboratory tests consisted of whole blood count, blood chemistry and electrolyte analysis, coagulation tests, and kidney function evaluation. Following data collection, two physicians were responsible for data verification, with a third researcher adjudicating any discrepancies in interpretation between them, if necessary.

To assess the status and severity of illness at the time of ICU admission, the Acute Physiology and Chronic Health Evaluation II (APACHE II), Charlson comorbidity index, and sequential organ failure assessment (SOFA) scores were calculated. Daily data on mechanical ventilation, AKI stages, and patient mortality were collected from electronic medical records.

CRRT-associated hemorrhagic events and electrolyte disturbances were investigated within the first 72 h of initiation. Hemorrhagic events included minor or major bleeding episodes, blood transfusion, low platelet count, and extended APTT or prothrombin time (PT) values, mmol/l), hyperkalemia (serum potassium > 1 g/dl in 24 h, while a minor hypophosphatemia (serum phosphorus < 1 g/dl) 24 h. Electrolyte disturbances involved hypokalemia (serum potassium < 3.5 mmol/l), hypernatremia (serum potassium > 5.5 mmol/l), hypocalcemia (serum ionized calcium < 2.5 Â mmol/l), hyperphosphatemia (serum phosphorus > 6.0 mg/dl), hyponatremia (serum sodium < 130 mmol/l), hypernatremia (serum sodium > 150 mmol/l), hypocalcemia (serum ionized calcium < 2.25 mmol/l), hypercalcemia (serum ionized calcium > 2.75 mmol/l), alkalosis (arterial pH > 7.45), and acidosis (arterial pH < 7.35).

### Outcomes

All eligible patients were retrospectively followed up from ICU admission to hospital discharge. The primary outcome was mortality rate. The mortality in the primary outcome was measured at the hospital discharge, referring to the total number of deaths during the follow-up period. The secondary outcomes included CRRT liberation, kidney recovery at hospital discharge (serum creatinine was two times higher than the baseline), the stages of AKI at ICU and hospital discharge, and mechanical ventilation in the ICU.

### Statistics

Continuous variables were summarized as median (IQR) and categorical variables as n (%). The Mann–Whitney U test was used to compare continuous variables between the high-altitude and low-altitude groups, or between the early CRRT initiation and delayed CRRT initiation groups, while the χ^2^ test or Fisher’s exact test was used to compare categorical variables. Kaplan–Meier curves were used to compare the survival rates of patients in the high- and low-altitude groups, as well as the early CRRT initiation and delayed CRRT initiation groups. At the same time, the corresponding 95% confidence intervals (Cis) and *P* values were calculated using the log-rank test. The association between the altitude or timing of CRRT initiation and the cumulative probability of kidney recovery at hospital discharge was analyzed using the Cox proportional hazards regression model, followed by adjustments of 13 cofounders identified through clinical experiences, literature review, and univariate analyses^22^. The 13 confounders included age, sex, ethnicity, BMI, altitude or timing of CRRT initiation, death, Charlson comorbidity index, APACHE II scores, SOFA scores, mechanical ventilation duration, CRRT duration, ICU duration, and hospitalization duration. The cause-specific “hazard” of recovery was then modeled. Statistical analyses were performed using SPSS Version 22.0. Two-tailed *P* values less than 0.05 were considered statistically significant unless otherwise specified. R version 4.1.0 was employed for the visualization of Kaplan–Meier curves and Cox model analysis.

## Results

### Demographic and clinical characteristics of patients treated with CRRT in high-altitude and low-altitude groups

After screening, 1124 eligible patients were identified and analyzed. A flowchart of the participant enrollment and grouping process is shown in Fig. 1. Of the 1124 patients, 648 patients (57.7%) from places equal to or above 3000 m were assigned to the high-altitude group and 476 patients (42.3%) from places below 3000 m were assigned to the low-altitude group. Although no differences were observed in sex, ethnicity, or BMI between the high group and low-altitude group (*P* > 0.05), patients in the high-altitude group were significantly younger (*P* < 0.001) (Table 1). The average ICU duration was 8.9 (IQR 4.1–18.5), and 538 patients (47.9%) died during the study. We observed a significantly higher proportion of SARS-CoV-2 infection among AKI patients in the high-altitude group compared to the low-altitude group (*P* = 0.002).

**Table 1:** Baseline characteristics of patients in high-altitude and low-altitude groups.

	Number (%)			*P* value
	Total (n = 1124)	High-altitude group (n = 648)	Low-altitude group (n = 476)	
Age (IQR)	55.2 (45.6–66.7)	54.1 (43.6–64.5)	59.6 (47.1–70.0)	< 0.001
Sex				0.85
Male	683 (60.8)	392 (60.5)	291 (61.1)	
Female	441 (39.2)	256 (39.5)	185 (38.9)	
Ethnicity				0.86
Han	498 (44.3)	289 (44.6)	209 (43.9)	
Tibetan	626 (55.7)	359 (55.4)	267 (56.1)	
BMI				0.41
< 18.5	65 (5.8)	39 (6.0)	26 (5.5)	
18.5–23.9	193 (17.2)	120 (18.5)	73 (15.3)	
24.0–27.9	482 (42.8)	278 (42.9)	204 (42.9)	
≥ 28.0	384 (34.2)	211 (32.6)	173 (36.3)	
Categories of patients				
Surgical	683 (60.8)	379 (58.5)	304 (63.9)	0.073
Sepsis	559 (49.7)	316 (48.8)	243 (51.1)	0.47
SARS-CoV-2	218 (19.4)	146 (22.5)	72 (15.1)	0.002
Laboratory results				
Serum creatinine (μmol/l) (IQR)	313.3 (229.6–485.3)	316.7 (235.2–507.9)	308.3 (216.7–475.0)	0.074
Serum BUN (mmol/l) (IQR)	6.8 (5.4–8.7)	6.8 (5.5–8.9)	6.6 (5.3–8.5)	0.15
Creatinine clearance (ml/min) (IQR)	38.9 (29.3–49.1)	34.9 (25.6–45.1)	47.4 (35.8–56.5)	< 0.001
Total bilirubin (μmol/l) (IQR)	16.1 (10.6–28.3)	16.5 (11.8–29.6)	14.9 (9.0–27.7)	0.27
Leukocytes, × 10^9^/l (IQR)	17.2 (12.2–26.1)	17.3 (12.7–26.6)	16.9 (11.1–24.9)	0.097
Platelet count, × 10^9^/l (IQR)	203.4 (121.7–322.8)	188.7 (104.3–307.9)	246.2 (153.7–367.5)	< 0.001
AKI stages				0.67
Stages 1	185 (16.5)	107 (16.5)	78 (16.4)	
Stages 2	360 (32.0)	214 (33.0)	146 (30.7)	
Stages 3	579 (51.5)	327 (50.5)	252 (52.9)	
CRRT modality				0.11
CVVHD	425 (37.8)	232 (35.8)	193 (40.5)	
CVVHDF	699 (62.2)	416 (64.2)	283 (59.5)	
Anticoagulation strategies				0.38
Regional citrate anticoagulation	713 (63.4)	404 (62.3)	309 (65.0)	
Unfractionated heparin	411 (36.6)	244 (37.7)	167 (35.0)	
Charlson comorbidity index (IQR)	4 (2–6)	4 (2–7)	4 (2–6)	0.35
APACHE II score (IQR)	27.0 (20.5–40)	28.5 (21.0–40.8)	25.0 (19.0–38.8)	0.098
SOFA score (IQR)	10 (8–12)	10 (8–12)	9 (7–12)	0.46
PaO_2_/FiO_2_ ratio (IQR)	166.4 (119.9–252.1)	161.7 (114.0–247.2)	176.3 (128.5–261.4)	0.075
Sedation	751 (66.8)	413 (63.7)	338 (71.0)	0.012
Mechanical ventilation	973 (86.6)	578 (89.2)	395 (83.0)	0.003
Mechanical ventilation duration (days) (IQR)	6.6 (2.5–14.8)	7.4 (3.5–15.9)	5.7 (1.8–13.2)	< 0.001
CRRT duration (days) (IQR)	4.4 (3.7–6.6)	4.8 (3.9–6.7)	3.7 (3.0–5.9)	0.036
ICU duration (days) (IQR)	8.9 (4.1–18.5)	10.5 (4.7–20.1)	7.2 (3.1–15.8)	0.007
Hospitalization duration (days) (IQR)	24.6 (15.6–38.8)	27.4 (18.8–41.5)	18.9 (10.3–35.5)	< 0.001

Data are shown by median (IQR) or n (%).

*AKI* acute kidney injury, *APACHE II* The Acute Physiology and Chronic Health Evaluation II, *BMI* body mass index, *BUN* blood urea nitrogen, *CRRT* Continuous renal replacement therapy, *CVVHD* continuous venovenous hemodialysis, *CVVHDF* continuous venovenous hemodiafiltration, *ICU* intensive care unit; *IQR* interquartile range, *PaO*_*2*_*/FiO*_*2*_ arterial oxygen tension/fraction of inspired oxygen, *SOFA* sequential organ failure assessment.

*P* values comparing between the groups are from χ^2^ test, Fisher’s exact test, or Mann–Whitney U test.

The laboratory results at the ICU admission indicated that creatinine clearance (CCr) (34.9 ml/min vs. 47.4 ml/min, *P* < 0.001) and platelet count (PLT) (188.7 × 10^9^/l vs. 246.2 × 10^9^/l, *P* < 0.001) of high-altitude group were significantly lower than that of low-altitude group (Table 1). However, there were no differences in the distribution of AKI stages (*P* = 0.67), CRRT modality application (*P* = 0.11), or anticoagulation strategies (*P* = 0.38) between the two groups. During the hospitalization, high-altitude patients received longer treatment of mechanical ventilation or CRRT and had a longer duration in ICU (10.5 days vs. 7.2 days, *P* = 0.007) or hospital (27.4 days vs. 18.9 days, *P* < 0.001) compared to the low-altitude patients, in spite of similar severities of illness in Charlson Comorbidity Index (4 vs. 4, *P* = 0.35), Acute Physiology and Chronic Health Evaluation II (APACHE II) score (28.5 vs. 25.0, *P* = 0.098), and Sequential Organ Failure Assessment (SOFA) score (10 vs. 9, *P* = 0.46) (Table 1).

### Kidney-related outcomes and CRRT related adverse events in high-altitude and low-altitude groups

The clinical characteristics and kidney-related outcomes of patients in both the high- and low-altitude groups are shown in Table 2. Throughout the study, progressively higher mortality was observed in the high-altitude group (*P* < 0.05). Compared to the low-altitude group, patients in the high-altitude group experienced a higher level of AKD staging at CRRT liberation (*P* = 0.033), ICU (*P* < 0.001), and hospital discharge (*P* < 0.001), but no changes at 24 h after CRRT initiation (*P* = 0.96). Meanwhile, more frequent mechanical ventilation was required in the high-altitude group at 24 h after CRRT initiation (64.4% vs. 51.7%, *P* < 0.001) and CRRT liberation (424.4% vs. 25.0%, *P* < 0.001). At 24 h after CRRT initiation, the PaO_2_/FiO_2_ ratio (193.6 vs. 229.3, *P* = 0.024), serum creatinine (126.3 μmol/l vs. 87.3 μmol/l, *P* = 0.007), CCr (33.2 ml/min vs. 41.8 ml/min, *P* < 0.001), serum blood urea nitrogen (BUN) (4.4 mmol/l vs. 3.1 mmol/l, *P* = 0.025), and total bilirubin (32.6 μmol/l vs. 26.5 μmol/l, *P* = 0.039) were significantly worse in high-altitude patients. However, the changes of serum creatinine (81.6 μmol/l vs. 77.2 μmol/l, *P* = 0.16) and BUN (2.9 mmol/l vs. 2.6 mmol/l, *P* = 0.42) were weakened at CRRT liberation.

**Table 2:** Clinical characteristics and kidney-related outcomes of patients in high-altitude and low-altitude groups.

	Number (%)			*P* value
	Total (n = 1124)	High-altitude group (n = 648)	Low-altitude group (n = 476)	
24 h after CRRT initiation				
AKD stages				0.96
Stages 0	12 (1.1)	6 (1.0)	6 (1.3)	
Stages 1	89 (7.9)	51 (7.9)	38 (8.0)	
Stages 2	135 (12.0)	75 (11.5)	60 (12.6)	
Stages 3	605 (53.8)	337 (52.0)	268 (56.3)	
Mechanical ventilation	663 (59.0)	417 (64.4)	246 (51.7)	< 0.001
PaO_2_/FiO_2_ ratio (IQR)	204.8 (146.9–298.0)	193.6 (137.1–289.6)	229.3 (166.2–315.4)	0.024
Serum creatinine (μmol/l) (IQR)	107.7 (57.3–184.2)	126.3 (61.8–217.9)	87.3 (41.9–169.5)	0.007
Creatinine clearance (ml/min) (IQR)	35.9 (26.4–48.7)	33.2 (24.7–45.8)	41.8 (30.6–53.0)	< 0.001
Serum BUN (mmol/l) (IQR)	4.0 (2.8–6.1)	4.4 (3.1–6.8)	3.1 (1.6–5.3)	0.025
Total bilirubin (μmol/l) (IQR)	30.1 (23.0–43.6)	32.6 (24.7–45.2)	26.5 (19.6–41.3)	0.039
Mortality	283 (25.2)	179 (27.6)	104 (21.8)	0.031
CRRT liberation				
AKD stages				0.033
Stages 0	23 (2.0)	12 (1.9)	11 (2.3)	
Stages 1	86 (7.7)	47 (7.3)	39 (8.2)	
Stages 2	172 (15.3)	76 (11.7)	96 (20.2)	
Stages 3	448 (39.9)	257 (39.6)	191 (40.1)	
Mechanical ventilation	394 (35.1)	275 (42.4)	119 (25.0)	< 0.001
PaO_2_/FiO_2_ ratio (IQR)	281.5 (200.4–399.7)	276.1 (187.8–394.5)	297.4 (221.0–401.2)	0.047
Serum creatinine (μmol/l) (IQR)	80.1 (35.4–166.3)	81.6 (37.9–184.1)	77.2 (32.8–142.6)	0.16
Serum BUN (mmol/l) (IQR)	2.9 (1.2–5.2)	2.9 (1.3–5.2)	2.6 (1.0–5.0)	0.42
Creatinine clearance (ml/min) (IQR)	42.1 (32.6–55.8)	40.7 (31.0–52.4)	47.9 (35.4–61.3)	0.017
Total bilirubin (μmol/l) (IQR)	39.2 (26.1–52.4)	41.6 (28.7–55.3)	32.9 (21.7–46.2)	0.016
CRRT reinstitution	181 (16.1)	117 (18.1)	64 (13.4)	< 0.001
Mortality	395 (35.1)	256 (39.5)	139 (29.2)	< 0.001
ICU discharge				
AKD stages				< 0.001
Stages 0	104 (9.3)	58 (9.0)	46 (9.7)	
Stages 1	97 (8.6)	25 (3.6)	72 (15.1)	
Stages 2	110 (9.8)	52 (8.2)	58 (12.2)	
Stages 3	327 (29.1)	206 (31.8)	121 (25.4)	
Mortality	486 (43.2)	307 (47.4)	179 (37.6)	< 0.001
Hospital discharge				
AKD stages				< 0.001
Stages 0	350 (31.1)	168 (25.9)	182 (38.2)	
Stages 1	87 (7.6)	31 (4.8)	56 (11.8)	
Stages 2	86 (7.6)	47 (7.3)	39 (8.2)	
Stages 3	63 (5.8)	53 (8.1)	10 (2.1)	
Mortality	538 (47.9)	349 (53.9)	189 (39.7)	< 0.001

Data are shown by median (IQR) or n (%).

*AKD* acute kidney diseases, *BUN* blood urea nitrogen, *CRRT* continuous renal replacement therapy, *ICU* intensive care unit, *IQR* interquartile range, *PaO2/FiO2* arterial oxygen tension/fraction of inspired oxygen.

P values comparing between the groups are from χ^2^ test, Fisher’s exact test, or Mann–Whitney U test.

Based on the data of Tables 1 and 2, compared to low-altitude group, high-altitude patients undergoing CRRT showed worse kidney-related outcomes, including more mechanical ventilation usage at ICU admission (89.2% vs. 83.0%, *P* = 0.003), 24 h after CRRT initiation (64.4% vs. 51.7%, *P* < 0.001) and CRRT liberation (424.4% vs. 25.0%, *P* < 0.001); longer durations of mechanical ventilation (7.4 days vs. 5.7 days, *P* < 0.001), CRRT (4.8 days vs. 3.7 days, *P* = 0.036), ICU (10.5 days vs. 7.2 days, *P* = 0.007) and hospitalization (27.4 days vs. 18.9 days, *P* < 0.001); lower incidence of AKD stage 0 at CRRT liberation (1.9% vs. 2.3%, *P* = 0.033), ICU (9.0% vs. 9.7%, *P* < 0.001) and hospital discharge (25.9% vs. 38.2%, *P* < 0.001); higher possibility of CRRT reinstitution after first liberation attempt (18.1% vs. 13.4%, *P* < 0.001); as well as higher mortality at 24 h after CRRT initiation (27.6% vs. 21.8%, *P* = 0.031), CRRT liberation (39.5% vs. 29.2%, *P* < 0.001), ICU (47.4% vs. 37.6%, *P* < 0.001) and hospital discharge (53.9% vs. 39.7%, *P* < 0.001).

Both high-altitude and low-altitude groups had a low incidence of CRRT-related adverse events (Supplementary Table 1). Notably, the differences were not significant in terms of minor (9.1% vs. 6.5%, *P* = 0.12) or major (16.8% vs. 14.3%, *P* = 0.28) bleeding episodes between the two groups. Although the high-altitude group had lower PLT (188.7 × 10^9^/l vs. 246.2 × 10^9^/l, *P* < 0.001) at the ICU admission (Table 1), the percentages of patients with PLT < 100 × 10^9^/l of the two groups (33.5% vs. 28.4%, *P* = 0.069) were similar within the first 72 h after CRRT initiation. Owing to the high efficiency of body hemostasis in CVVHDF and CVVHD, differences in major electrolyte disturbances were not observed between these two groups (*P* > 0.05).

### Impacts of altitude to AKI survival and renal recovery

Early after randomization, the survival curves diverged in favor of the high-altitude group and remained separated afterwards (log-rank test, *P* < 0.001) (Supplementary Fig. 1). Moreover, patients in the low-altitude group spent more time outside the ICU (10.5 days vs. 7.2 days, *P* = 0.007) (Table 1).

The Cox model was used to assess relevant factors affecting the cumulative probability of renal recovery across the entire cohort. After adjusting for confounders, an independent relationship between altitude and renal recovery was discovered (hazard ratio, 0.36; 95% CI 0.24 to 0.56; *P* < 0.001) (Supplementary Table 4). Simultaneously, death was also correlated with renal recovery (hazard ratio, 1.18; 95% CI, 1.03 to 1.27; *P* = 0.003) (Supplementary Table 4). Even after accounting for death as a competing risk factor for kidney recovery, the cumulative probability of kidney recovery in the low-altitude group was still higher than that in the high-altitude group (*P* < 0.001) (Supplementary Fig. 2).

### Contributions of early CRRT initiation to survival and renal outcomes in high altitudes

Subsequently, high-altitude patients (n = 648) were divided into an early CRRT initiation group (n = 406) and a delayed CRRT initiation group (n = 242) based on the time of CRRT initiation to investigate the optimal timing of CRRT initiation. A total of 476 low-altitude patients were divided into an early CRRT initiation group (n = 291) and a delayed CRRT initiation group (n = 185) (Fig. 1). For high-altitude patients, the median time from AKI diagnosis to CRRT initiation in the early group was significantly shorter than that in the delayed group (16.5 h vs. 34.5 h, *P* = 0.004). Early CRRT initiation group had better kidney-related outcomes than delayed CRRT initiation group, including more rapid recovery of laboratory results of PaO_2_/FiO_2_ ratio (189.2 vs. 254.5, *P* = 0.007), serum creatinine (54.2 μmol/l vs. 109.5 μmol/l, *P* < 0.001), CCr (45.1 min vs. 37.6 ml/min, *P* < 0.001), BUN (2.0 mmol/l vs. 4.1 mmol/l, *P* = 0.002) and total bilirubin (34.2 μmol/l vs. 46.3 μmol/l, *P* = 0.003) at CRRT liberation; shorter durations of mechanical ventilation (5.8 days vs. 9.9 days, *P* = 0.028), CRRT (3.5 days vs. 6.3 days, *P* = 0.014), ICU (7.6 days vs. 14.3 days, *P* = 0.006) and hospitalization (20.7 days vs. 31.2 days, *P* < 0.001); higher incidence of AKD stage 0 at CRRT liberation (2.2% vs. 1.2%, *P* = 0.026), ICU (9.6% vs. 7.9%, *P* < 0.001) and hospital discharge (33.7% vs. 12.8%, *P* < 0.001); lower possibility of CRRT reinstitution after first liberation attempt (12.8% vs. 26.9%, *P* < 0.001) (Table 3, Supplementary Table 2). However, no differences were observed between the early and delayed CRRT initiation groups in the low-altitude patients.

**Table 3:** Clinical characteristics and kidney-related outcomes of patients in early CRRT and delayed CRRT initiation groups.

	High-altitude group (n = 648)		*P* value	Low-altitude group (n = 476)		*P* value
	Early CRRT initiation group (n = 406)	Delayed CRRT initiation group (n = 242)		Early CRRT initiation group (n = 291)	Delayed CRRT initiation group (n = 185)	
24 h after CRRT initiation						
AKD stages			0.48			0.38
Stages 0	4 (1.0)	2 (0.8)		3 (1.0)	3 (1.6)	
Stages 1	38 (9.4)	13 (5.4)		33 (11.3)	15 (8.1)	
Stages 2	49 ( 12.1)	26 (10.8)		32 (11.0)	28 (15.1)	
Stages 3	213 (52.4)	124 (51.2)		160 (55.1)	98 (53.0)	
Mechanical ventilation	259 (63.8)	158 (65.3)	0.74	154 (52.9)	92 (49.7)	0.50
PaO_2_/FiO_2_ ratio (IQR)	206.2 (157.3–314.5)	181.8 (114.0–252.9)	0.085	234.1 (182.5–327.6)	218.7 (158.8–298.4)	0.098
Serum creatinine (μmol/l) (IQR)	104.5 (39.8–187.6)	159.7 (101.1–243.9)	< 0.001	81.3 (35.9–152.4)	95.0 (52.7–181.2)	0.17
Creatinine clearance (ml/min) (IQR)	40.6 (28.5–50.1)	29.3 (20.7–41.4)	< 0.001	42.4 (32.5–58.0)	39.5 (28.3–46.8)	0.23
Serum BUN (mmol/l) (IQR)	3.5 (2.3–5.9)	5.2 (3.9–7.7)	0.004	3.0 (1.4–5.0)	3.5 (1.9–5.8)	0.11
Total bilirubin (μmol/l) (IQR)	31.8 (22.9–43.5)	33.2 (26.1–48.4)	0.17	25.7 (17.8–38.1)	27.4 (21.3–43.9)	0.13
Mortality	102 (25.1)	77 (31.8)	0.070	63 (21.6)	41 (22.2)	0.91
CRRT liberation						
AKD stages			0.026			0.46
Stages 0	9 (2.2)	3 (1.2)		7 (2.4)	5 (2.7)	
Stages 1	33 (8.1)	14 (5.8)		24 (8.2)	15 (8.1)	
Stages 2	60 (14.7)	16 (6.6)		46 (15.8)	40 (21.6)	
Stages 3	157 (38.8)	100 (41.4)		127 (43.7)	73 (39.5)	
Mechanical ventilation	153 (37.7)	122 (50.4)	< 0.001	68 (23.4)	51 (27.6)	0.35
PaO_2_/FiO_2_ ratio (IQR)	289.2 (196.7–401.3)	254.5 (168.3–377.6)	0.007	305.2 (233.8–415.5)	289.7 (210.6–391.4)	0.089
Serum creatinine (μmol/l) (IQR)	54.2 (21.7–168.3)	109.5 (50.2–224.8)	< 0.001	75.9 (31.7–138.6)	81.1 (33.5–151.9)	0.095
Serum BUN (mmol/l) (IQR)	2.0 (1.0–3.9)	4.1 (2.0–6.8)	0.002	2.5 (1.0–4.6)	2.8 (1.0–5.5)	0.10
Creatinine clearance (ml/min) (IQR)	45.1 (36.3–62.7)	37.6 (26.8–48.4)	< 0.001	48.1 (36.9–62.5)	46.8 (35.1–59.7)	0.14
Total bilirubin (μmol/l) (IQR)	34.2 (22.8–46.5)	46.3 (32.7–60.8)	0.003	31.8 (20.4–44.9)	33.5 (23.2–48.0)	0.093
CRRT reinstitution	52 (12.8)	65 (26.9)	< 0.001	35 (12.0)	29 (15.7)	0.32
Mortality	147 (36.2)	109 (45.0)	0.031	87 (29.9)	52 (28.1)	0.76
ICU discharge						
AKD stages			< 0.001			0.75
Stages 0	39 (9.6)	19 (7.9)		27 (9.3)	19 (10.3)	
Stages 1	18 (4.4)	7 (2.9)		41 (14.1)	31 (16.8)	
Stages 2	48 (11.8)	4 (1.7)		35 (12.0)	23 (12.4)	
Stages 3	123 (30.4)	83 (34.2)		78 (26.8)	43 (23.2)	
Mortality	178 (43.8)	129 (53.3)	0.023	110 (37.8)	69 (37.3)	0.92
Hospital discharge						
AKD stages			< 0.001			0.69
Stages 0	137 (33.7)	31 (12.8)		111 (38.1)	71 (38.4)	
Stages 1	22 (5.4)	9 (3.7)		34 (11.7)	22 (11.9)	
Stages 2	34 (8.4)	13 (5.4)		24 (8.2)	15 (8.1)	
Stages 3	11 (2.7)	42 (17.4)		8 (2.8)	2 (1.1)	
Mortality	202 (49.8)	147 (60.7)	0.007	114 (39.2)	75 (40.5)	0.78

Data are shown by median (IQR) or n (%).

*AKD* acute kidney diseases, *BUN* blood urea nitrogen, *CRRT* continuous renal replacement therapy, *ICU* intensive care unit, *IQR* interquartile range, *PaO2/FiO2* arterial oxygen tension/fraction of inspired oxygen.

P values comparing between the groups are from χ^2^ test, Fisher’s exact test, or Mann–Whitney U test.

Supplementary Table 3 summarizes the incidence of CRRT-related adverse events in the early and delayed CRRT initiation groups. For high-altitude patients, both minor (8.4% vs. 10.3%, *P* = 0.40) or major (15.0% vs. 19.3%, *P* = 0.13) bleeding episodes and coagulation functions of PLT (32.2% vs. 35.5%, *P* = 0.44), APTT (40.8 s vs. 45.4 s, *P* = 0.074) or PT (21.8 s vs. 27.4 s, *P* = 0.098) were comparable. However, lower incidences of hypokalemia (6.9% vs. 14.0%, *P* = 0.004), hyperkalemia (9.1% vs. 17.4%, *P* = 0.003), hyperphosphatemia (10.8% vs. 19.0%, *P* = 0.005), hyponatremia (5.2% vs. 10.7%, *P* = 0.012), hypocalcemia (28.8% vs. 42.1%, *P* = 0.001), and acidosis (11.8% vs. 19.0%, *P* = 0.015) were observed in the early CRRT initiation group.

**Figure 2:**
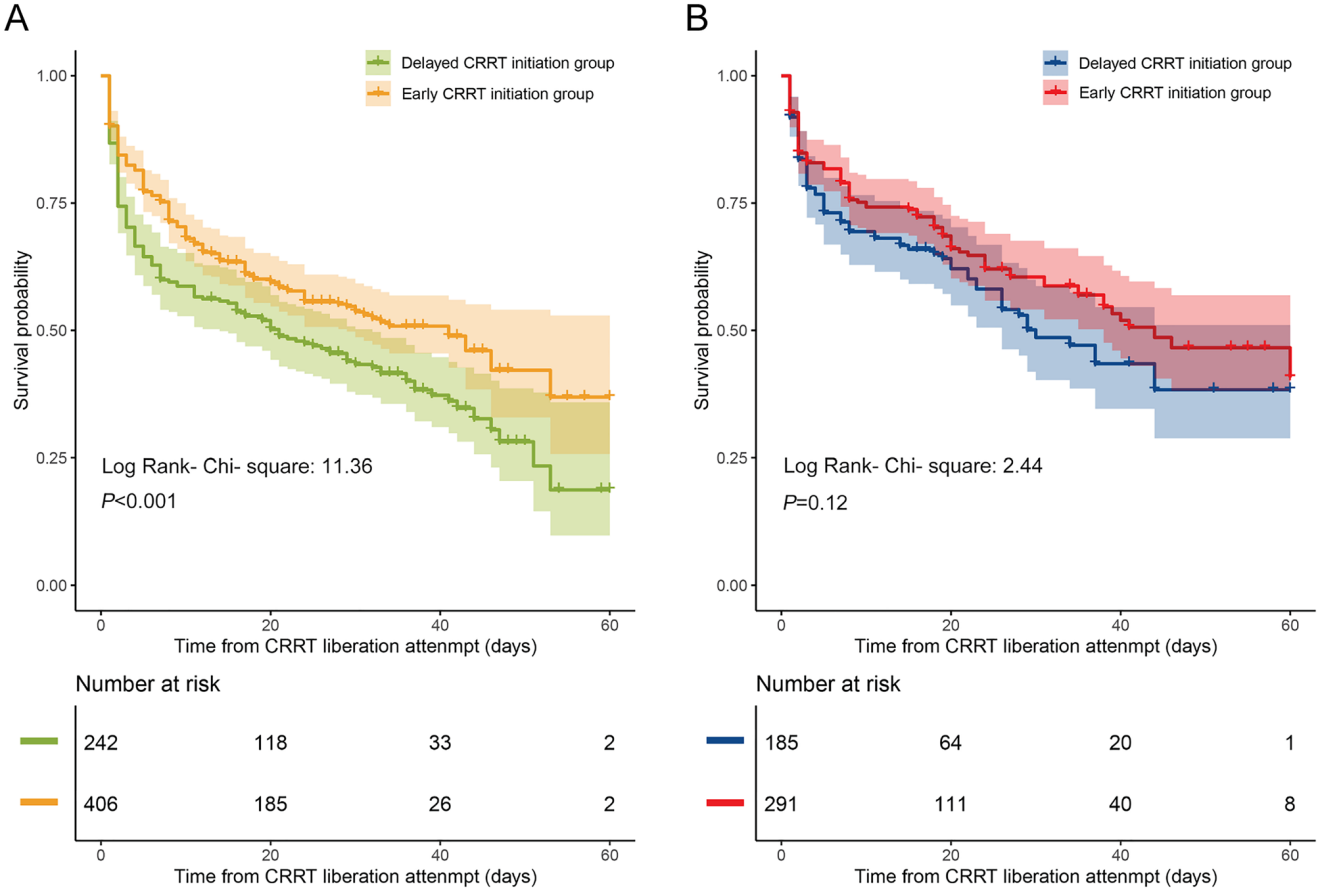
Kaplan–Meier survival plots for early continuous renal replacement therapy (CRRT) initiation group and delayed CRRT initiation group in high-altitude patients (**A**) and low-altitude patients (**B**), respectively. The corresponding 95% confidence intervals (CIs) of the two groups were calculated and marked, respectively. Log-rank test was employed to compare the survival curves.

**Figure 3:**
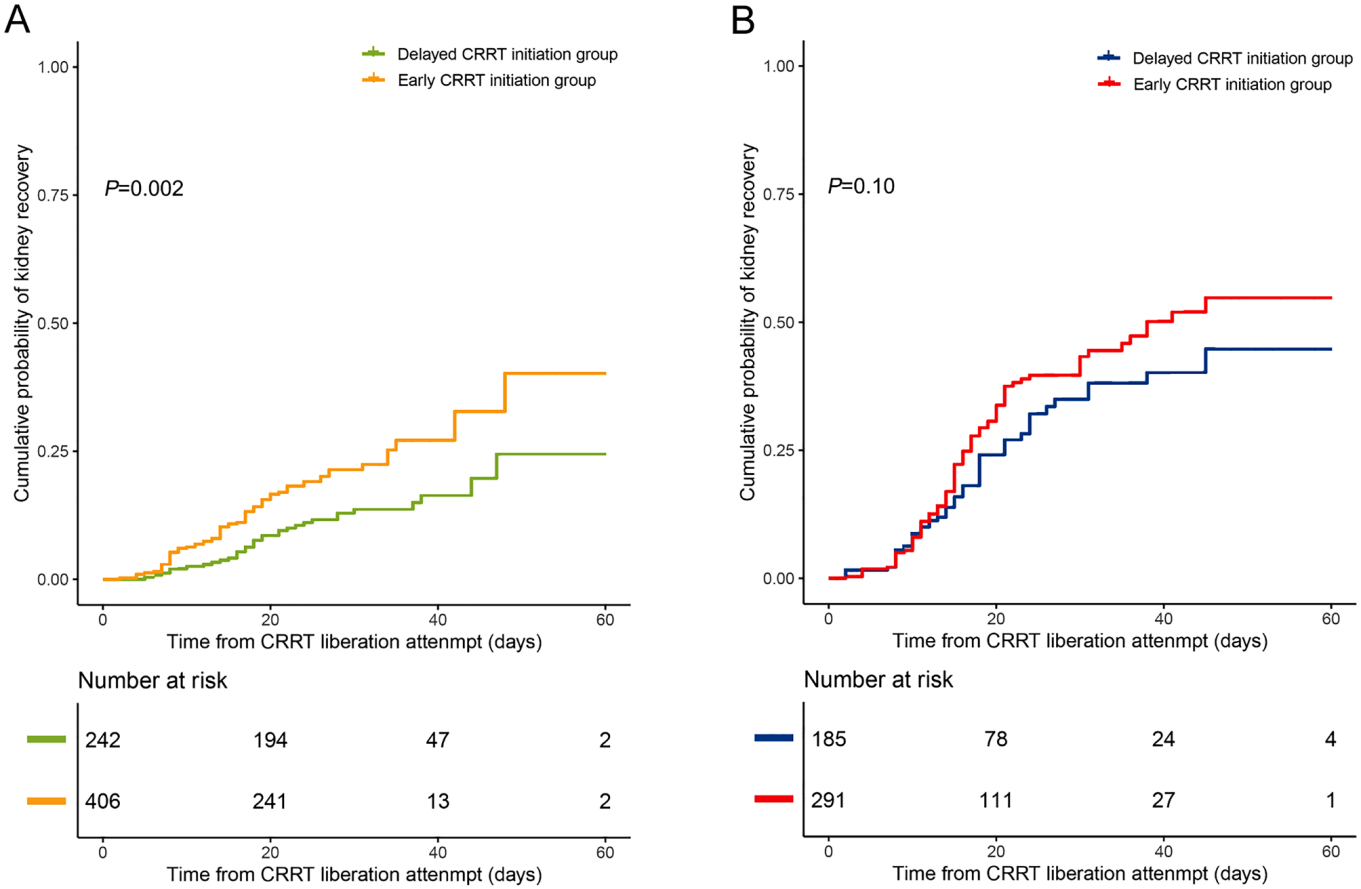
The cumulative probability of kidney recovery after continuous renal replacement therapy (CRRT) liberation attempt for early CRRT initiation and delayed CRRT initiation groups in high-altitude patients (**A**) and low-altitude patients (**B**), respectively.

Consistent with the lower mortality in the early CRRT initiation group at CRRT liberation (36.2% vs. 45.0%, *P* = 0.031), ICU (43.8% vs. 53.3%, *P* = 0.023), and hospital discharge (49.8% vs. 60.7%, *P* = 0.007) (Table 3) of high-altitude patients, Kaplan–Meier curve analysis also indicated significant survival differences between the two groups (log-rank chi-square test, *P* < 0.001) (Fig. 2). Nevertheless, early CRRT did not seem to influence the survival rate of low-altitude patients (*P* = 0.12). Meanwhile, a positive association between CRRT initiation and renal recovery was observed in the high-altitude group after adjusting for confounders (hazard ratio, 0.25; 95% CI, 0.15 to 0.43; *P* < 0.001) (Supplementary Table 5). Likewise, the cumulative probability of kidney recovery was also significantly higher in the early CRRT initiation group (*P* = 0.002) (Fig. 3). However, this relationship was unlikely to be applicable to the low-altitude group (Fig. 3, Supplementary Table 6).

## Discussion

In this retrospective, multi-center cohort study, our findings indicated worse clinical outcomes in patients treated with CRRT for AKI at high altitudes. Compared to low-altitude patients, high-altitude patients had a higher mortality rate but a lower rate of kidney recovery. Altitude was found to be independently correlated with the cumulative probability of kidney recovery. Despite the fact that critically ill patients at high altitudes have fewer platelets and more coagulation dysfunction, CRRT for AKI is not likely to increase bleeding risk or other adverse events. In contrast, Kaplan–Meier curves and Cox model analysis revealed that early CRRT initiation may reduce mortality and promote renal recovery at high altitudes.

Numerous studies have reported on the poor kidney-related outcomes of critically ill patients with AKI in the ICU^2,23–26^. A meta-analysis of 24 observational studies of trauma patients in the ICU found that the incidence of post-traumatic AKI was 24% and the risk of death was 3.4 times higher than that in patients without AKI^26^. Meanwhile, a recent multinational study of over 1800 patients from 97 ICUs in America demonstrated that 57% of patients developed AKI within one week after admission, with mortality rates ranging from 40 to 55%, revealing that AKI was an independent risk factor for death in the ICU^24,25^. Our findings are consistent with those of the previous studies. During the observation period, the overall mortality rate was 47.9%. High-altitude patients died at a significantly higher rate than low-altitude patients (53.9% vs. 39.7%, *P* < 0.001). However, only 31.1% of the patients reached stage 0 AKD and achieved kidney recovery at the end of the study, which differed slightly from previous studies^2,25^.

The increased risk of AKI mortality at high altitudes may partly contribute to severe hypoxemia. Our study was conducted in Tibet, China, one of the highest places in the world, with an approximately 64% oxygen concentration at sea level^27^. Native highlanders suffered from hypoxia throughout the years. Several studies have demonstrated that hypoxia is directly related to the development of kidney injury symptoms, such as elevation of blood creatinine and urea, hematuria, proteinuria, and cytokines and cytokine storms^28–30^. Respiratory syndrome coronavirus 2 (SARS-CoV-2) causes body oxygen deficiency due to lung damage, leading to 5–23% of patients being diagnosed with AKI in the hospital and 68% in the ICU^31,32^. Due to the fact that our hospital only started conducting SARS-CoV-2 nucleic acid testing in 2020 and 2021, the number of patients in our study was relatively small. However, we still observed a significantly higher proportion of SARS-CoV-2 infection among AKI patients in the high-altitude group compared to the low-altitude group. This finding confirmed our initial hypothesis. The hypoxia caused by SARS-CoV-2 infection can exacerbate the condition of AKI patients in the high-altitude group. Although the precise mechanisms are unclear, the hypoxia-inducible factor (HIF) signaling pathway and reactive oxygen species (ROS), resulting in regulated cell death of renal tubular cells, may play an important role in AKI^33^. In our study, differences in oxygenation function between the high- and low-altitude groups were in accordance with that of mortality. Although no changes in the PaO_2_/FiO_2_ ratio were found at ICU admission between the two groups (*P* = 0.075), high-altitude patients had a slower recovery of the PaO_2_/FiO_2_ ratio at 24 h after CRRT initiation (*P* = 0.024) and CRRT liberation (*P* = 0.047). Two possible reasons were speculated for the PaO_2_/FiO_2_ ratio consistency at ICU admission: on the one hand, chronic high-altitude exposure resulted in many positive adaptations involving the stability of the PaO_2_/FiO_2_ ratio in native highlanders^27^; on the other hand, the use of sedatives upon ICU admission was significantly higher in the low-altitude group. Thus, we speculated that the similarity in PaO_2_/FiO_2_ between the two groups may be attributed to the utilization of sedative therapy upon ICU admission^34^. However, changes in the PaO_2_/FiO_2_ ratio were not entirely consistent with the mechanical ventilation results. In our study, high-altitude patients were likely to require more frequent and longer mechanical ventilation, indicating that these patients had persistent hypoxia. Further research into the pathogenesis and mechanisms of hypoxemia and AKI mortality at high altitudes is critical for understanding this phenomenon.

Polycythemia in highlanders is another possible pathogenetic mechanism underlying the higher mortality of AKI at high altitudes. Excess erythrocytosis, characterized by hemoglobin (Hb) > 190 g/l, was found to be more common in native highlanders living at altitudes above 2500 m^6^. Long-term exposure to hypoxic environments at high altitudes would upregulate the expression of HIF-2α, resulting in polycythemia with high blood viscosity and a remarkable decrease in renal plasma flow^6,35^. When the increased filtration fraction fails to compensate for the reduction in chronic hypoxia, mechanical stress-induced kidney injury occurs^6,33^. As a result, persistent kidney injury and death from AKI are more likely to occur in high-altitude areas.

CRRT has been widely used for the management of critically ill patients in the ICU. However, the incidences of CRRT-related adverse effects were highly scrutinized and needed to be reduced to promote CRRT quality and patient safety^36^. The main concerns regarding CRRT are bleeding risk and electrolyte loss. Anticoagulation therapy, including heparin and citrate anticoagulation, was applied to prevent filter overclotting, but at the expense of increased bleeding risk^36^. Notably, bleeding risks at high altitudes might be more dangerous because various reports have described detrimental coagulation disorders there^37^. However, our findings revealed no differences in bleeding risks or blood transfusion requirements between the high- and low-altitude groups.

Our findings also indicate the difficulty in kidney recovery at high altitudes after AKI. Based on the results of the Cox model analysis, patients at high altitudes experienced a lower cumulative probability of kidney recovery than those at low altitudes (*P* < 0.001). The kidney of AKI, unlike chronic kidney disease (CKD), may resolve to some extent, although the course may range from several days to years^1,38^. A prospective cohort study proved that early recovery of kidney function is closely linked to better long-term outcomes in AKI-D^39^. Our results are consistent with those of a previous study. The high-altitude group’s early recovery of kidney function was also lower at 24 h after CRRT initiation (*P* < 0.05). Studies have found that CRRT could assist in improving renal function, alleviating renal burden, and providing continuous supportive treatment in critically ill patients with hemodynamic instability and AKI^40^. However, CRRT did not completely offset the effects of altitude on the outcomes of patients with AKI. There were two possible reasons for the failed kidney recovery at high altitudes. The first is the persistent existence of uncorrectable hypoxia. Despite mechanical ventilation, long-term high-altitude exposure keeps the body under hypoxic conditions, resulting in continuous kidney damage^29,30,41^. The second reason was the poorer condition of the kidney at high altitudes. High-altitude renal syndrome impeded the kidney function recovery of these patients^6,17^.

The optimal timing of CRRT initiation in critically ill AKI patients has long been debated. Considering economic benefits and possibility of renal function recovery in absence of CRRT, a watchful waiting strategy for those patients of AKI without the life-threatening hyperkalemia or hemodynamics dysfunction was confirmed safe and effective^2,42^. However, many nephrologists advocate the early initiation of CRRT in the ICU owing to its potential advantages in fluid control, inflammatory cytokine removal, body homeostasis, and severe complications prevention^23,43,44^. There are many landmark clinical trials focusing on the issue of CRRT initiation timing. The Effect of Early vs Delayed Initiation of Renal Replacement Therapy on Mortality in Critically Ill Patients With Acute Kidney Injury (ELAIN) trial was a single-center, non-blinded RCT in Germany. Among 231 patients with stages 2 AKI, those in the early initiation group (n = 112) started RRT promptly (median time from diagnosis to CRRT initiation of 6 h), while the delayed initiation group (n = 119) started CRRT only when absolute indications or progression to stages 3 AKI occurred (median time from diagnosis to CRRT initiation of 25.5 h). The 90-day all-cause mortality rate was 39.3% in the early initiation group vs. 54.7% in the delayed initiation group^20^. However, the Artificial Kidney Initiation in Kidney Injury (AKIKI) trial enrolled 619 stages 3 AKI patients who were randomized to immediate initiation of CRRT (median time from diagnosis to CRRT initiation of 4.3 h) or a delayed initiation strategy (median time from diagnosis to CRRT initiation of 57 h). The 60-day mortality rate did not differ between the groups (48.5% vs. 49.7%; *P* = 0.79)^45^. A more recently completed Initiation of Dialysis Early Versus Delayed in the Intensive Care Unit (IDEAL-ICU) trial included 488 septic AKI patients. Patients without urgent indications for CRRT were randomized to early initiation (n = 246) or delayed initiation (n = 242). The 90-day mortality rate was 58% in the early initiation group vs. 54% in the delayed initiation group (*P* = 0.38)^21^.

However, according to our findings, early CRRT initiation may contribute to a higher rate of survival and kidney recovery at high altitudes than at low altitudes. Moreover, early initiation of CRRT at high altitudes also resulted in a lower incidence of CRRT-related adverse events. The heterogeneity of the study populations was one of the possible reasons for the advantages of early CRRT initiation in our study. Among the patients included in our study, the high-altitude group had a generally younger age compared to the low-altitude group. The age difference between the two groups might also introduce bias in the results. Younger AKI patients in the high-altitude group were more likely to achieve renal function recovery through CRRT. However, this also reflected that patients at high altitudes were more susceptible to AKI. In contrast to previous studies, we focused on the high-altitude population in this study. As mentioned previously, high-altitude patients with AKI had worse outcomes and faster progression, which appeared to make early CRRT initiation more beneficial^2,23,24^. Moreover, electrolyte disorders and cytokine imbalances resulting from hypoxia tend to occur in high-altitude patients, leading to severe kidney damage coupled with AKI^6,8,9^. Thus, the elimination of redundant products by early CRRT might provide an opportunity for kidney rest and repair in these patients. However, further research is required to validate this hypothesis. Our results offer at least one of the efficient ways to solve the increased mortality of critically ill patients with AKI at high altitudes.

Our study has several limitations. First, although a large number of cases and time points had been designed to predict the outcomes of AKI, a 60 days’ follow-up might not be long enough to detect differences in long-term survival or cumulative probability of renal recovery between groups. In fact, follow-up after hospital discharge might be more meaningful for determining kidney-related outcomes. However, data for this retrospective study were difficult to obtain. Second, the majority of the baseline serum creatinine values in our study were unavailable. As a result, we attempted to estimate the baseline values using the converted serum creatinine level as an alternative for AKI-D diagnosis and staging. However, the possibility of bias in our study must be acknowledged. Third, due to the inherent flaws of retrospective studies, there were no completely predefined standard criteria for CRRT practices before the study. We could not exclude the possibility of physicians making empirical decisions regarding CRRT initiation, termination, or reinstitution. A larger prospective study is required to confirm our findings. Fourth, other criteria have been used to define the timing of CRRT initiation, such as BUN level, urine output, and AKI staging. Only the time from diagnosis to CRRT initiation in this study might have affected the results.

## Conclusions

In this retrospective multi-center cohort study, we suggest worse clinical outcomes in patients undergoing CRRT for AKI at high altitudes. Compared with low-altitude patients, high-altitude patients experience higher mortality and worse kidney-related outcomes. Altitude was independently correlated with the cumulative probability of kidney recovery. Despite fewer platelets and coagulation dysfunction in critically ill patients at high altitudes, CRRT for AKI was unlikely to increase the risk of bleeding and other adverse events. Conversely, the results of Kaplan–Meier curves and Cox model analysis showed that early CRRT initiation might decrease mortality and promote renal recovery in high-altitude patients.

### Supplementary Information

Supplementary Information.

## Data Availability

The original contributions of this study are included in the article or Supplementary Information. Further inquiries can be directed to the corresponding author.
