# Supplementary material for: The benefits of early continuous renal replacement therapy in critically ill patients with acute kidney injury at high-altitude areas: a retrospective multi-center cohort study
Source: Sci Rep. 2023 Sep 9;13:14882. doi: 10.1038/s41598-023-42003-6 (PMC10492831; doi:10.1038/s41598-023-42003-6)
Supplement: Supplementary file 1 — Supplementary Information. [file 41598_2023_42003_MOESM1_ESM.docx]

| Supplementary table 1. Adverse events of CRRT in high-altitude and low-altitude groups | | | | |
| --- | --- | --- | --- | --- |
|  | Number (%) | | | *P* value |
|  | Total (n=1,124) | High-altitude group (n=648) | Low-altitude group (n=476) |  |
| Minor bleeding episode | 90 (8.0) | 59 (9.1) | 31 (6.5) | 0.12 |
| Major bleeding episode | 177 (15.7) | 109 (16.8) | 68 (14.3) | 0.28 |
| Blood transfusion | 254 (22.6) | 158 (24.4) | 96 (20.2) | 0.097 |
| Platelet count <100×10^9^/L | 352 (31.3) | 217 (33.5) | 135 (28.4) | 0.069 |
| Peak APTT in the first 72 h of CRRT (IQR) | 41.4 (29.3-58.7) | 42.3 (29.9-60.1) | 39.7 (28.2-56.6) | 0.11 |
| Peak PT in the first 72 h of CRRT (IQR) | 23.0 (15.4-35.6) | 23.6 (15.7-36.8) | 21.4 (14.9-33.5) | 0.24 |
| Hypokalemia | 110 (9.8) | 62 (9.6) | 48 (10.1) | 0.84 |
| Hyperkalemia | 131 (11.7) | 79 (12.2) | 52 (10.9) | 0.57 |
| Hypophosphatemia | 243 (21.6) | 135 (20.8) | 108 (22.7) | 0.46 |
| Hyperphosphatemia | 155 (13.8) | 90 (13.9) | 65 (13.7) | 0.93 |
| Hyponatremia | 68 (6.0) | 47 (7.3) | 21 (4.4) | 0.057 |
| Hypernatremia | 25 (2.2) | 15 (2.3) | 10 (2.1) | 0.84 |
| Hypocalcemia | 382 (34.0) | 219 (33.8) | 163 (34.2) | 0.90 |
| Hypercalcemia | 312 (27.8) | 174 (26.9) | 138 (29.0) | 0.46 |
| Alkalosis | 324 (28.8) | 176 (27.2) | 148 (31.1) | 0.16 |
| Acidosis | 172 (15.3) | 94 (14.5) | 78 (16.4) | 0.40 |

Data are shown by median (IQR) or n (%). *P* values comparing between the groups are from χ^2^ test, Fisher's exact test, or Mann-Whitney U test. APTT: Activated partial thromboplastin time; CRRT: Continuous renal replacement therapy; IQR: Interquartile range; PT: Prothrombin time.

| Supplementary table 2. Baseline characteristics of patients in early CRRT and delayed CRRT initiation groups | | | | | | |
| --- | --- | --- | --- | --- | --- | --- |
|  | High-altitude group (n=648) | | *P* value | Low-altitude group (n=476) | | *P* value |
|  | Early CRRT initiation group (n=406) | Delayed CRRT initiation group (n=242) |  | Early CRRT initiation group (n=291) | Delayed CRRT initiation group (n=185) |  |
| Age (IQR) | 53.5 (40.B5:G24 | 57.2 (45.8-59.0) | 0.084 | 58.6 (46.2-71.5) | 61.9 (49.0-73.8) | 0.12 |
| **Sex** |  |  | 0.74 |  |  | 0.63 |
| Male | 248 (61.1) | 144 (59.5) |  | 175 (60.1) | 116 (62.7) |  |
| Female | 158 (38.9) | 98 (40.5) |  | 116 (39.9) | 69 (37.3) |  |
| **Ethnicity** |  |  | 0.33 |  |  | 0.26 |
| Han | 175 (43.1) | 114 (47.1) |  | 134 (46.0) | 75 (40.5) |  |
| Tibetan | 231 (56.9) | 128 (52.9) |  | 157 (54.0) | 110 (59.5) |  |
| **BMI** |  |  | 0.67 |  |  | 0.42 |
| <18.5 | 27 (6.7) | 12 (5.0) |  | 17 (5.8) | 9 (4.9) |  |
| 18.5-23.9 | 78 (19.2) | 42 (17.4) |  | 46 (15.8) | 27 (14.6) |  |
| 24.0-27.9 | 174 (42.8) | 104 (43.0) |  | 116 (39.9) | 88 (47.6) |  |
| ≥28.0 | 127 (31.3) | 84 (34.7) |  | 112 (38.5) | 61 (32.9) |  |
| **Categories of patients** |  |  |  |  |  |  |
| Surgical | 227 (55.9) | 152 (62.8) | 0.099 | 186 (63.9) | 118 (63.8) | >0.99 |
| Sepsis | 194 (47.8) | 122 (50.4) | 0.57 | 147 (50.5) | 96 (51.9) | 0.78 |
| SARS-CoV-2 | 69 (17.0) | 77 (31.8) | <0.001 | 44 (15.1) | 28 (15.1) | >0.99 |
| **Laboratory results** |  |  |  |  |  |  |
| Serum creatinine (μmol/l) (IQR) | 308.2 (219.7-456.9) | 335.0 (256.6-549.3) | 0.072 | 299.6 (194.5-449.2) | 318.1 (237.4-524.9) | 0.094 |
| Serum BUN (mmol/l) (IQR) | 6.2 (5.0-8.5) | 7.5 (5.9-9.6) | 0.034 | 6.4 (4.9-8.0) | 7.0 (5.6-8.9) | 0.17 |
| Creatinine clearance (ml/min) (IQR) | 34.2 (24.9-43.7) | 36.0 (27.8-46.4) | 0.16 | 44.2 (31.8-54.3) | 50.2 (41.7-59.4) | 0.087 |
| Total bilirubin (μmol/l) (IQR) | 17.1 (12.6-32.5) | 14.9 (8.9-25.1) | 0.28 | 16.5 (10.8-33.6) | 13.8 (7.6-23.4) | 0.34 |
| Leukocytes, ×10^9^/L (IQR) | 18.9 (14.6-28.1) | 15.7 (10.0-25.5) | 0.098 | 17.5 (12.0-26.5) | 15.4 (10.8-21.9) | 0.15 |
| Platelet count, ×10^9^/L (IQR) | 196.2 (110.8-325.7) | 185.3 (98.9-287.5) | 0.37 | 251.5 (169.3-393.2) | 239.4 (137.9-329.0) | 0.28 |
| **AKI stages** |  |  | 0.53 |  |  | 0.98 |
| Stages 1 | 62 (15.3) | 45 (18.6) |  | 47 (16.2) | 31 (16.8) |  |
| Stages 2 | 135 (33.3) | 79 (32.6) |  | 89 (30.6) | 57 (30.8) |  |
| Stages 3 | 209 (51.4) | 118 (48.8) |  | 155 (53.2) | 97 (52.4) |  |
| **CRRT modality** |  |  | 0.80 |  |  | 0.63 |
| CVVHD | 147 (36.2) | 85 (35.1) |  | 121 (41.6) | 72 (38.9) |  |
| CVVHDF | 259 (63.8) | 157 (64.9) |  | 170 (58.4) | 113 (61.1) |  |
| **Anticoagulation strategies** |  |  | 0.45 |  |  | 0.33 |
| Regional citrate anticoagulation | 258 (63.5) | 146 (60.3) |  | 194 (66.7) | 115 (62.2) |  |
| Unfractionated heparin | 148 (36.5) | 96 (39.7) |  | 97 (33.3) | 70 (37.8) |  |
| Charlson Comorbidity Index (IQR) | 16.5 (13.0-21.5) | 34.5 (28.5-41.5) | 0.004 | 15.8 (11.9-20.8) | 35.8 (29.2-42.4) | 0.003 |
| APACHE Ⅱ score (IQR) | 4 (2-6) | 4 (2-8) | 0.85 | 4 (2-7) | 4 (2-6) | 0.89 |
| SOFA score (IQR) | 26.0 (18.7-39.0) | 29.5 (22.7-42.6) | 0.14 | 24.5 (18.0-37.5) | 26.0 (20.5-40.5) | 0.21 |
| PaO_2_/FiO_2_ ratio (IQR) | 10 (7-12) | 11 (9-13) | 0.11 | 10 (8-14) | 8 (6-11) | 0.074 |
| Mechanical ventilation | 170.4 (125.3-272.6) | 147.2 (100.3-224.9) | 0.095 | 175.8 (104.2-271.6) | 171.0 (138.5-259.1) | 0.26 |
| Mechanical ventilation duration (days) (IQR) | 365 (89.9) | 213 (88.0) | 0.51 | 247 (84.9) | 148 (80.0) | 0.17 |
| CRRT duration (days) (IQR) | 5.8 (2.3-10.6) | 9.9 (6.0-18.2) | 0.028 | 5.4 (1.6-12.3) | 6.2 (2.4-15.8) | 0.18 |
| ICU duration (days) (IQR) | 3.5 (2.1-5.3) | 6.3 (4.3-8.6) | 0.014 | 3.2 (2.7-5.6) | 3.9 (3.1-6.4) | 0.11 |
| Hospitalization duration (days) (IQR) | 7.6 (3.8-16.2) | 14.3 (8.5-23.6) | 0.006 | 6.8 (3.0-14.7) | 7.6 (3.5-16.6) | 0.23 |

Data are shown by median (IQR) or n (%). *P* values comparing between the groups are from χ^2^ test, Fisher's exact test, or Mann-Whitney U test. AKI: Acute kidney injury; APACHE Ⅱ: The Acute Physiology and Chronic Health Evaluation Ⅱ; BMI: Body mass index; BUN: blood urea nitrogen; CRRT: Continuous renal replacement therapy; CVVHD: Continuous venovenous hemodialysis; CVVHDF: Continuous venovenous hemodiafiltration; ICU: Intensive care unit; IQR: Interquartile range; PaO_2_/FiO_2_: Arterial oxygen tension/fraction of inspired oxygen; SOFA: Sequential Organ Failure Assessment.

| Supplementary table 3. Adverse events in patients undergoing CRRT in early CRRT and delayed CRRT initiation groups | | | | | | |
| --- | --- | --- | --- | --- | --- | --- |
|  | High-altitude group (n=648) | | *P* value | Low-altitude group (n=476) | | *P* value |
|  | Early CRRT initiation group (n=406) | Delayed CRRT initiation group (n=242) |  | Early CRRT initiation group (n=291) | Delayed CRRT initiation group (n=185) |  |
| Minor bleeding episode | 34 (8.4) | 25 (10.3) | 0.40 | 18 (6.2) | 13 (4.2) | 0.71 |
| Major bleeding episode | 61 (15.0) | 48 (19.8) | 0.13 | 39 (13.4) | 29 (15.7) | 0.50 |
| Blood transfusion | 96 (23.6) | 62 (25.6) | 0.57 | 57 (19.6) | 39 (21.1) | 0.91 |
| Platelet count <100×10^9^/L | 131 (32.2) | 86 (35.5) | 0.44 | 81 (27.8) | 54 (29.2) | 0.76 |
| Peak APTT in the first 72 h of CRRT (IQR) | 40.8 (26.5-56.7) | 45.4 (33.9-67.6) | 0.074 | 38.1 (26.8-53.5) | 41.3 (30.7-59.1) | 0.18 |
| Peak PT in the first 72 h of CRRT (IQR) | 21.8 (14.0-32.5) | 27.4 (17.5-41.0) | 0.098 | 20.7 (14.2-32.5) | 21.9 (15.5-34.8) | 0.64 |
| Hypokalemia | 28 (6.9) | 34 (14.0) | 0.004 | 27 (9.3) | 21 (11.4) | 0.53 |
| Hyperkalemia | 37 (9.1) | 42 (17.4) | 0.003 | 28 (9.6) | 24 (13.0) | 0.29 |
| Hypophosphatemia | 80 (19.7) | 55(22.7) | 0.37 | 62 (21.3) | 46 (24.9) | 0.37 |
| Hyperphosphatemia | 44 (10.8) | 46 (19.0) | 0.005 | 38 (13.1) | 27 (14.6) | 0.68 |
| Hyponatremia | 21 (5.2) | 26 (10.7) | 0.012 | 11 (3.8) | 10 (5.4) | 0.49 |
| Hypernatremia | 8 (2.0) | 7 (2.9) | 0.59 | 6 (2.1) | 4 (2.2) | 0.99 |
| Hypocalcemia | 117 (28.8) | 102 (42.1) | 0.001 | 96 (33.0) | 67 (36.2) | 0.49 |
| Hypercalcemia | 101 (24.9) | 73 (30.2) | 0.14 | 78 (26.8) | 60 (32.4) | 0.21 |
| Alkalosis | 107 (26.4) | 69 (28.5) | 0.58 | 81 (27.8) | 67 (36.2) | 0.067 |
| Acidosis | 48 (11.8) | 46 (19.0) | 0.015 | 42 (14.4) | 36 (19.5) | 0.16 |

Data are shown by median (IQR) or n (%). *P* values comparing between the groups are from χ^2^ test, Fisher's exact test, or Mann-Whitney U test. APTT: Activated partial thromboplastin time; CRRT: Continuous renal replacement therapy; IQR: Interquartile range; PT: Prothrombin time.

| Supplementary table 4. Multivariate Cox regression model for the cumulative probability of kidney recovery after CRRT liberation attempt in high-altitude and low-altitude groups | | |
| --- | --- | --- |
| Factors | Number (n=1,124) | *P* value |
|  | HR (95% CI) |  |
| Altitude (high altitude vs low altitude) | 0.36 (0.24-0.56) | <0.001 |
| **Adjustment covariate** |  |  |
| Age | 0.68 (0.53-0.76) | 0.29 |
| Sex | 0.72 (0.58-0.85) | 0.31 |
| Ethnicity | 0.88 (0.60-1.01) | 0.35 |
| BMI | 0.84 (0.56-1.22) | 0.43 |
| Death | 1.18 (1.03-1.27) | 0.003 |
| Charlson Comorbidity Index | 1.00 (0.92-1.08) | 0.86 |
| APACHE Ⅱ scores | 0.93 (0.70-1.14) | 0.29 |
| SOFA scores | 0.79 (0.48-1.02) | 0.14 |
| Mechanical ventilation duration | 0.92 (0.69-1.18) | 0.19 |
| CRRT duration | 2.02 (0.99-3.98) | 0.061 |
| ICU duration | 0.90 (0.69-1.10) | 0.77 |
| Hospitalization duration | 0.81 (0.52-1.19) | 0.34 |

AKI: Acute kidney injury; APACHE Ⅱ: The Acute Physiology and Chronic Health Evaluation Ⅱ; BMI: Body mass index; CI: Confidence intervals; CRRT: Continuous renal replacement therapy; HR: Hazard ratio; ICU: Intensive care unit; SOFA: Sequential Organ Failure Assessment.

| Supplementary table 5. Multivariate Cox regression model for the cumulative probability of kidney recovery after CRRT liberation attempt of high-altitude patients in early CRRT and delayed CRRT initiation groups | | |
| --- | --- | --- |
| Factors | Number (n=1,124) | *P* value |
|  | HR (95% CI) |  |
| Altitude (high altitude vs low altitude) | 0.25 (0.15-0.43) | <0.001 |
| **Adjustment covariate** |  |  |
| Age | 0.63 (0.42-0.87) | 0.49 |
| Sex | 0.89 (0.63-1.05) | 0.36 |
| Ethnicity | 0.81 (0.58-1.02) | 0.43 |
| BMI | 0.74 (0.49-0.98) | 0.45 |
| Death | 1.13 (1.01-1.25) | <0.001 |
| Charlson Comorbidity Index | 1.10 (0.94-1.37) | 0.17 |
| APACHE Ⅱ scores | 0.92 (0.75-1.18) | 0.24 |
| SOFA scores | 0.98 (0.81-1.24) | 0.19 |
| Mechanical ventilation duration | 0.65 (0.38-0.91) | 0.32 |
| CRRT duration | 1.28 (1.01-1.54) | 0.087 |
| ICU duration | 1.01 (0.75-1.30) | 0.098 |
| Hospitalization duration | 0.86 (0.57-1.19) | 0.33 |

AKI: Acute kidney injury; APACHE Ⅱ: The Acute Physiology and Chronic Health Evaluation Ⅱ; BMI: Body mass index; CI: Confidence intervals; CRRT: Continuous renal replacement therapy; HR: Hazard ratio; ICU: Intensive care unit; SOFA: Sequential Organ Failure Assessment.

| Supplementary table 6. Multivariate Cox regression model for the cumulative probability of kidney recovery after CRRT liberation attempt of low-altitude patients in early CRRT and delayed CRRT initiation groups | | |
| --- | --- | --- |
| Factors | Number (n=1,124) | *P* value |
|  | HR (95% CI) |  |
| Altitude (high altitude vs low altitude) | 0.74 (0.48-0.98) | 0.43 |
| **Adjustment covariate** |  |  |
| Age | 0.58 (0.35-0.80) | 0.27 |
| Sex | 0.66 (0.40-0.85) | 0.47 |
| Ethnicity | 1.11 (0.96-1.21) | 0.094 |
| BMI | 1.05 (0.79-1.34) | 0.10 |
| Death | 1.15 (1.08-1.34) | 0.002 |
| Charlson Comorbidity Index | 0.82 (0.59-1.05) | 0.35 |
| APACHE Ⅱ scores | 0.79 (0.50-1.02) | 0.39 |
| SOFA scores | 0.69 (0.46-0.91) | 0.41 |
| Mechanical ventilation duration | 0.88 (0.61-1.02) | 0.36 |
| CRRT duration | 1.25 (1.03-1.56) | 0.091 |
| ICU duration | 0.94 (0.77-1.19) | 0.21 |
| Hospitalization duration | 1.04 (0.76-1.31) | 0.11 |

AKI: Acute kidney injury; APACHE Ⅱ: The Acute Physiology and Chronic Health Evaluation Ⅱ; BMI: Body mass index; CI: Confidence intervals; CRRT: Continuous renal replacement therapy; HR: Hazard ratio; ICU: Intensive care unit; SOFA: Sequential Organ Failure Assessment.


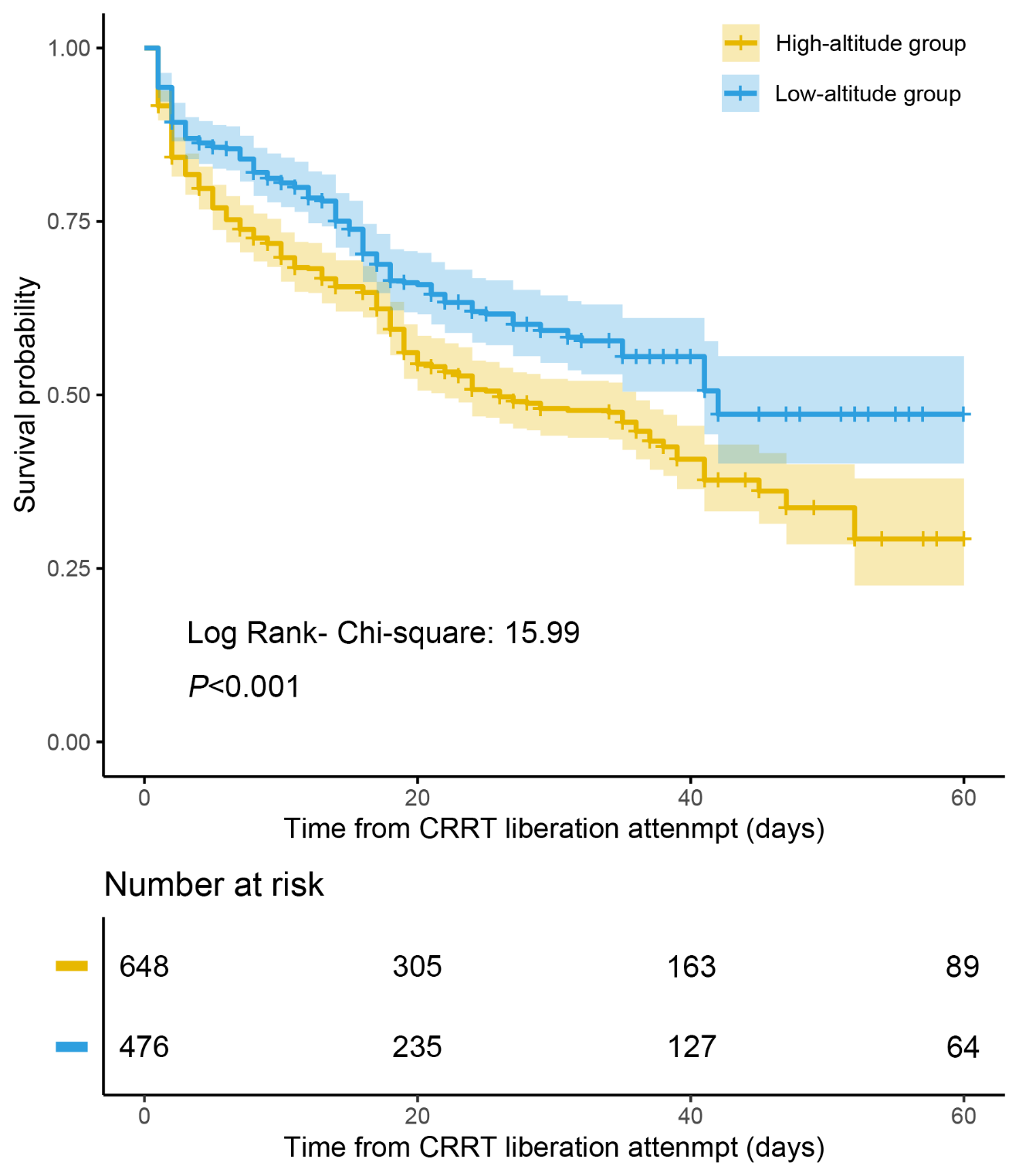


Supplementary figure 1. Kaplan–Meier survival plots for high-altitude group and low-altitude group. The corresponding 95% confidence intervals (CIs) of the two groups were calculated and marked, respectively. Log-rank test was employed to compare the survival curves.


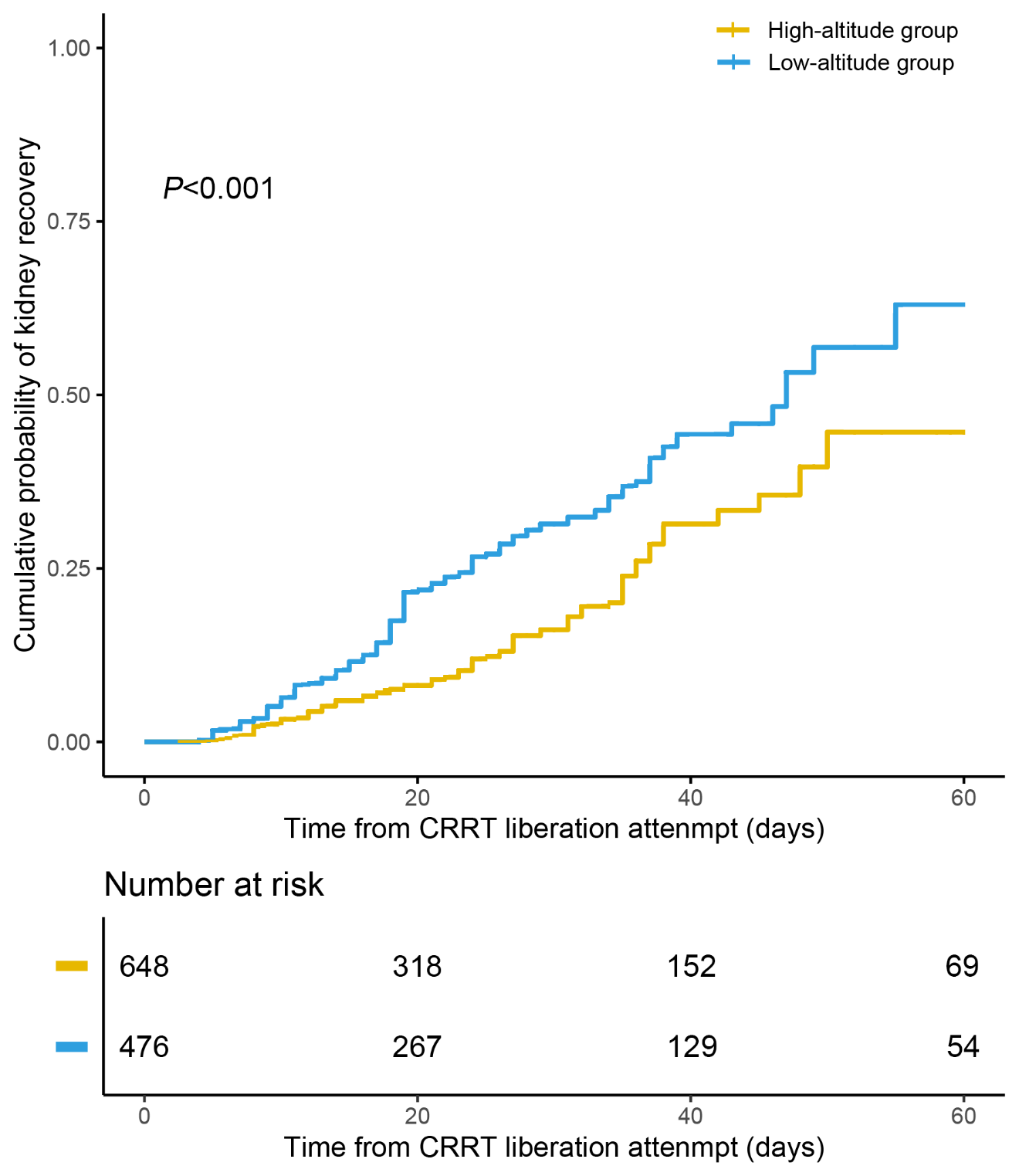


Supplementary figure 2. The cumulative probability of kidney recovery after continuous renal replacement therapy (CRRT) liberation attempt for high-altitude group and low-altitude group.
